# Editorial: Unconventional Animal Models in Infectious Disease Research – Part I

**DOI:** 10.3389/fcimb.2021.759621

**Published:** 2021-11-29

**Authors:** Nadin Younes, Gheyath K. Nasrallah

**Affiliations:** ^1^ Biomedical Research Center, Qatar University Health, Qatar University, Doha, Qatar; ^2^ Department of Biomedical Science, College of Health Sciences, Qatar University Health, Qatar University, Doha, Qatar

**Keywords:** vaccines,, therapeutic agents,, infectious disease,, host-pathogen interactions,, animal model

The multiplicity of host-pathogen interactions associated with infectious diseases frequently necessitate the use of a complicated biological system. Therefore, animal models have contributed substantially to unraveling the physiopathology of infectious diseases. The feasibility to manipulate different animal models aids in (i) discovering the role of host or microbial factors in the infection pathogenesis, (ii) understanding different mechanisms of tissue invasion, host defense, pathogen dissemination. This Research Topic, which includes 8 high-quality research papers (7 original research articles, and 1 review), sheds the light on the use of unconventional model as a platform for advancement in infectious disease research and address novel ground-breaking findings in host-pathogen interaction studies, which paved the way for discovering new therapeutic targets and vaccine development. The most important findings are summarized in **Table 1**.

**Table 1:** Summary for the outcomes of all accepted articles in this special issue.

Study type	Organism Name	Animal Model	Aim	Methods of assessment	Conclusion	Reference
Original article	DTMUV virus	Duck	To study the role of autophagy in facilitating DTMUV replication “*in vivo*”	• Western blot (WB) • Hematoxylin and eosin staining • Immunohistochemistry • RT-PCR	• DTMUV trigged autophagy, which facilitates its replication inside the cells and induces pathological symptoms	Hu et al.
Original article	DEV virus	Duck	To elucidate the roles of US1 and its NLS in DEV replication “*in vivo*”	• RT-PCR • WB • Immunofluorescence assay	• The DEV US1 ORF is 990 bp • Molecular mass of the ICP22 protein is ~57 kDa • ICP22 contains a classical NLS at 305-312AA that is essential for its localization to the nucleus • DEV US1 is non-essential for host infection but associated with a severe growth deficit *in vitro.*	Li et al.
Original article	*M. fortuitum*	Zebrafish	To study the disease pathogenesis of *M. fortuitum* infections	• Creation of fluorescent *M. fortuitum* • Morpholino Injection and CFTR Knockdown • Zebrafish Microinjection and Infection • Zebrafish live imaging	• Zebrafish embryos form granulomas as early as 2 days post-infection • Transient macrophage depletion in zebrafish led to rapid embryo death with increased bacterial extracellular cord formation	Johansen and Kremer
Original article	*G. mellonella* larvae	*S. aureus*	To study the function of sRNAs during *S. aureus* infection “*in vivo*”	• Bacterial growth in the larvae • Immunohistochemistry • Bacterial isolation and RNA extraction • Monitoring RNA expression levels	• *G. mellonella* larvae is a suitable model to study sRNA-mediated pathogenesis in *S. aureus* • sprD and sprC increased during infection and associated with mortality • rnaIII expression remained barely detectable over time	Ménard et al.
Original article	*P.berghei*	*A.gambiae*	Study the function of CLIPB10 in protease cascades “*in vivo*” using A. gambiae as a model	• RT-PCR • Activation of recombinant zymogens • Substrate screening of active CLIPB10_Xa_ • MALDI-TOF MS Analysis	• *proPO* plays an essential role in the cuticular melanization in insects • CLIPB10 is required for the melanization of ookinete stages of the rodent malaria parasite *P. berghei.* • Recombinant serpin 2 protein formed a stable protein complex with CLIPB10 protein	Zhang et al.
Original article	*C. elegans*	*E. faecium*	To analyze a panel of lab strains of *E. faecium* with deletions of targeted virulence factor “*in vivo*” using C. *elegans* as a model	• *C. elegans* - Enterococcus pathogenesis assays • Colony forming unit assay • Genomic analysis • Microscopy • RNA interference protocol	• C. *elegans* is a high throughput infection model for studying the pathogensis of *E. facium* • Removal of certain virulence factors (e.g., Δfms15) was sufficient to affect the virulence of *E. faecium* • Multiple deletions were required to affect pathogenesis, suggesting that host-pathogen interactions are multifactorial.	Revtovich et al.
Original article	Mouse	*Helicobacter, Clostridium, Lactobacillus, Klebsiella, Rodentibacter and Enterococcus*	To study the effect of Autoinducer-2 on the process of necrotizing enterocolitis mouse model	• Histology • Immunohistochemistry • Mouse Intestinal Content Acquisition and AI-2 Activity Measurement • Fecal sample microbiota analysis ELISA RT-PCR-WB	• The AI-2 level was significantly decreased in the NEC group • In the NA (NEC + AI-2) group; the intestinal injury scores, expression of TLR4, NF-kB, and proinflammatory factors were reduced, and expression of anti-inflammatory factor was increased compared to NEC group • At the phylum level, the Proteobacteria abundance in the NA group was significantly increased. • At the genus level, *Helicobacter* and *Clostridium* exhibited significantly greater abundance in the NEC group compared to the other two groups	Ji et al.
Review	Drosophila	Different pathogens	Gaining proper insight into host–pathogen interactions using drosophila as a model	• Not applicable	• *In vivo* drosophila studies enabled the identification of humoral and cell-mediated host defense factors against a wide array of intracellular and extracellular pathogens	Younes et al.

The first article focused on the role of autophagy during duck tembusu virus (DTMUV) infection using duck as a model (Hu et al.). The authors used two drugs as autophagy regulators, which are rapamycin (autophagy enhancer) and methyladenine and chloroquine (autophagy inhibitor). They discovered that DTMUV infection triggered autophagy in duck’s spleen and brain. They also demonstrated that the autophagy inhibitors suppressed DTMUV replication and reduced DTMUV-induced pathogenic symptoms. They concluded that autophagic regulation was linked to the expression of innate immunity genes such as the pattern recognition receptors, type I interferons, and cytokines.

The second article also utilized ducks to study the role of DEV ICP22 protein in duck enteritis virus (DEV) (Li et al.). DEV duplicate US1 genes encode a DEV ICP22 protein. The authors reported that ICP22 protein, molecular mass of 57 kDa, can enter the nucleus by itself using conventional NLS motif. However, DEV ICP22 protein cannot enter the nucleus after mutating amino acid 309R, demonstrating that this amino acid is the crucial residue for ICP22 localization. Most importantly, they concluded that the DEV ICP22 protein is a non-essential immediate-early protein primarily found in the nucleus of infected duck-embryo-fibroblasts (DEF) cells and that US1 deletion can hamper DEV replication.

Johansen and Kremer were able to show that zebrafish embryos are susceptible to *M. fortuitum* infection in a dose-dependent manner. Interestingly, they showed that *M. fortuitum* was able to form granulomas in embryos as early as 2 days post-infection. In addition, they noticed that transient macrophage depletion led to rapid embryo death. Interestingly, they reported that the depletion of cystic fibrosis transmembrane conductance regulator (*CFTR)* in zebrafish by morpholino significantly increased the mortality rate, bacterial burden, and abscess formation. This is consistent with previous studies showing that CFTR expression on both innate and adaptive immune cells contributes to immunological dysfunction in cystic fibrosis (CF) (Hu et al.; Johansen and Kremer; Li et al.; Sermet-Gaudelus et al., 2003; Bruscia et al., 2009; Bonfield et al., 2012; Richards and Olivier, 2019).

Insect models proved to be very valuable for infectious diseases studies. Ménard et al., developed a non-mammalian infection model using larval *G. mellonella* to investigate the function of small regulatory RNAs (sRNAs) during *S. aureus* infection. The authors collected total RNA from *S. aureus* at several time points during the infection. They concluded that the expression patterns of the examined sRNAs were distinct and fluctuated over time, with small pathogenicity island RNA D (*sprD*) and small pathogenicity island RNA C (*sprC*) expression increasing during infection and correlated with larval death. In addition, they found out that when either *sprD* or *sprC* is deleted, the decline in insect death rates is delayed. The findings of Ménard et al. shed lights on the usefulness of *G. mellonella* as an infection model to investigate all 50 bona fide sRNAs known to be expressed by *S. aureus*.

Zhang et al., also utilized insects as a model to study the function of CLIPB10 in protease cascades “*in vivo*”. Activation of prophenoxidase (*proPO*), a form of immune system regulation, leads to the formation of eumelanin on foreign microorganisms. ProPO activation is tightly controlled by different mechanisms including clip domain serine proteases (CLIPs), proteolytically inactive homologs, and serpin inhibitors. The authors studied the function of the CLIPB10 in *Anopheles gambiae*, which is the most common malaria vector in Sub-Saharan Africa. They reported that CLIPB10 was required for the melanization of ookinete stages of *Plasmodium berghei*. In addition, recombinant serpin 2 protein, a key inhibitor of the *proPO* activation cascade, efficiently inhibited CLIPB10 activity *in vitro*. The authors concluded that CLIPB10 along with CLIPB9 plays a critical role as the second protease with prophenoloxidase-activating function in *A. gambiae* suggesting functional redundancy in the protease network that controls melanization.

Studying enterococcal pathogenesis generally requires vertebrates’ models, making them slow, expensive, and ethically problematic (Weiner et al., 2016). Therefore, Revtovich et al., developed the first high-throughput *Caenorhabditis elegans* infection model. *They* successfully showed that *E. faecium* could effectively invade and colonize the intestine of *C. elegans* and elicit an immunological response. They used this model to examine a panel of lab strains with targeted virulence factor deletions. Although deletion of a single virulent factor such as *Δfms15* was sufficient to affect virulence, multiple deletions were generally necessary to alter the pathogenesis, implying that host-pathogen interactions are multifactorial.

Autoinducer-2 (AI-2) is thought to be a bacterial interspecies signaling molecule that plays an important role in the physiological behaviors of bacteria. Ji et al., studied the role of AI-2 in Necrotizing enterocolitis (NEC) mouse model. They randomly divided C57BL/6 mice into three groups: control, NEC, and NEC+AI-2 (NA). They reported that AI-2 significantly decreased in the NEC group. In addition, the intestinal injury scores, expression of TLR4, NF‐kB, and proinflammatory factors were reduced in the NA group, whereas the expression of anti-inflammatory factors was increased in the NA group. They concluded that AI-2 partially reverses flora disorder and decreases inflammation in an NEC mouse model.

Younes et al., review discussed the use of drosophila in host–pathogen interaction research. Drosophila has recently gained a lot of attention due to the evolutionarily conserved features with higher vertebrates, such as cascades of the innate immune, pathways of signal transduction, and transcriptional regulators. The versatility, low cost, affordable maintenance, short life cycle, high fecundity, well-characterized genome made drosophila one of the most powerful model species (Younes et al.).

## Author Contributions

NY: drafted the editorial. GN: designed the work, revised the editorial critically for important intellectual content and provided final approval of the version to be published. All authors contributed to the article and approved the submitted version.

## Conflict of Interest

The authors declare that the research was conducted in the absence of any commercial or financial relationships that could be construed as a potential conflict of interest.

## Publisher’s Note

All claims expressed in this article are solely those of the authors and do not necessarily represent those of their affiliated organizations, or those of the publisher, the editors and the reviewers. Any product that may be evaluated in this article, or claim that may be made by its manufacturer, is not guaranteed or endorsed by the publisher.
